# Bridging the Practice Gap: Polycystic Ovary Syndrome as an Under-Recognised Risk State for Ischaemic Stroke and Cardiovascular Disease

**DOI:** 10.3390/jcm15103577

**Published:** 2026-05-07

**Authors:** Maryam Jamshaid, Ambreen Ali Sheikh, Warda Jamshaid, Hajra Shafiq, Mohamed H Ahmed

**Affiliations:** 1 North Mersey Stroke Service, Aintree University Hospital, Liverpool University Hospitals Foundation Trust, Liverpool L9 7AL, UK; ambreen.alisheikh@liverpoolft.nhs.uk; 2 Department of Acute Medicine, Russells Hall Hospital, Dudley Group NHS Foundation Trust, Dudley DY1 2HQ, UK; warda.jamshaid3@nhs.net; 3 Department of Obstetrics and Gynaecology, Glangwili Hospital, Hywel Dda University Health Board, Carmarthen SA31 2AF, UK; hajra.shafiq@wales.nhs.uk; 4 Department of Medicine for Older People and Department of Diabetes, North Cheshire and Mersey NHS Foundation Trust, Warrington WA5 1QG, UK; mohamed.hassan-ahmed@nhs.net; 5 Faculty of Medicine and Health Sciences, University of Buckingham, Buckingham MK18 1EG, UK

**Keywords:** polycystic ovary syndrome, PCOS, ischaemic stroke, cerebrovascular disease, stroke, cardiovascular disease, cardiometabolic risk, risk prediction, women’s health, endocrine disorders

## Abstract

Polycystic ovary syndrome (PCOS) is the most common endocrine disorder in reproductive-aged women. Epidemiologic evidence increasingly indicates that women with PCOS exhibit elevated risk of ischaemic stroke. Unlike prior reviews that have focused predominantly on general cardiovascular risk in PCOS, this review specifically examines ischaemic stroke risk, its mechanistic links, and the extent to which current stroke and cardiovascular frameworks fail to account for PCOS. We conducted a narrative review examining the association of PCOS on ischaemic stroke and cardiovascular disease (CVD). Large-scale cohort studies and meta-analyses from 2000 to 2025 were identified from Medline, Embase, PubMed, and Google Scholar, with specific emphasis on ischaemic stroke in women with PCOS. Case reports and case series were excluded. There is substantial evidence supporting the relationship between PCOS and increased vascular risk. Large-scale cohort studies and meta-analyses report higher rates of cerebrovascular events among women with PCOS. A recent 2025 meta-analysis pooling 11 studies reported a statistically significantly increased risk of stroke in women with PCOS (OR = 1.89, 95% CI = 1.22–2.55), although heterogeneity was high (I^2^ = 97.7%), indicating important variation in study design, populations, and confounder adjustment. Other meta-analyses report more modest associations, and some studies did not demonstrate significant associations with myocardial infarction or all-cause mortality. Several analyses also showed attenuation of risk following adjustment for body mass index and metabolic factors. The observed association between PCOS and vascular risk should be interpreted with caution, given substantial heterogeneity and potential confounding by cardiometabolic factors. These findings raise the possibility that increased risk may be mediated through established pathways such as obesity, insulin resistance, and dyslipidaemia rather than representing a consistently independent effect. Despite this, PCOS remains inconsistently recognised within clinical frameworks. Current stroke guidelines and widely used risk prediction tools, including QRISK3 and the Essen Stroke Risk Score, do not incorporate PCOS, highlighting a potential gap in sex-specific risk assessment. Evidence demonstrating that inclusion of PCOS improves risk prediction or clinical outcomes remains limited. However, under-recognition of vascular risk in women with PCOS may limit timely risk assessment and intervention. This review highlights a number of implementation gaps globally, including gaps in research and inter-disciplinary communication. Priority next steps include prospective studies to clarify whether PCOS independently contributes to vascular risk, and predictive modelling studies to determine whether including PCOS improves risk stratification. In parallel, multidisciplinary care pathways may support earlier identification and prevention in higher-risk women.

## 1. Introduction

Polycystic ovary syndrome (PCOS) is a multifaceted endocrine disorder affecting an estimated 4–20% of women during their reproductive years [1]. Its clinical presentation is diverse, reflecting underlying disturbances such as disordered follicular maturation, insulin resistance, and androgen excess [2]. Core features include menstrual cycle irregularities, subfertility, obesity, and biochemical hyperandrogenism [2]. In addition to reproductive manifestations, PCOS is strongly associated with metabolic dysfunction, encompassing dyslipidaemia, central obesity, hypertension, impaired glucose tolerance, and type II diabetes [3]. Collectively, these abnormalities accelerate atherosclerotic processes, increasing the risk of cardiovascular disease (CVD) [4].

Several diagnostic frameworks have been established for PCOS, the most widely recognised being the National Institutes of Health criteria (1990), the Rotterdam criteria (2003), and the Androgen Excess Society definition (2006) [5,6,7]. These multiple definitions give rise to four phenotypic subtypes: phenotype A (hyperandrogenism, ovulatory dysfunction, and polycystic ovarian morphology); phenotype B (hyperandrogenism with ovulatory dysfunction); phenotype C (ovulatory PCOS, defined by hyperandrogenism and polycystic ovarian morphology); and phenotype D (non-hyperandrogenic PCOS, with ovulatory dysfunction and polycystic ovarian morphology). Phenotypes A and B are considered classic PCOS; C and D are viewed as non-classic types. Cardiovascular risk appears highest among women with hyperandrogenic phenotypes; however, increased prevalence of cardiometabolic risk factors is observed across all phenotypic groups [8].

According to the 2025 World Health Organisation (WHO) report, CVD remains the leading cause of death worldwide, responsible for approximately 19.8 million deaths annually [9]. Of these CVD deaths, 85% were due to myocardial infarction and stroke, with a considerable proportion occurring before the age of 70 [9]. Obesity, which is highly prevalent in PCOS and may emerge in adolescence or earlier, is an independent driver of cardiovascular risk [4]. Furthermore, dyslipidaemia is present in up to 70% of affected women, typically characterised by reduced high-density lipoprotein (HDL) and elevated low-density lipoprotein (LDL) concentrations [10].

Epidemiologic data show that women with PCOS have higher rates of cardiovascular and cerebrovascular events, including increased risks of coronary heart disease and stroke. [11,12]. Although PCOS is well recognised for its reproductive and metabolic consequences, its potential relevance to cerebrovascular disease, particularly ischaemic stroke, remains less clearly defined and under-recognised in clinical practice. Key abnormalities in insulin signalling pathways, adipose tissue dysfunction, androgen metabolism and current or recent hormonal therapy confer an adverse vascular risk profile in women with PCOS [12].

Despite growing but heterogenous evidence, a practice gap persists. The 2023 National Clinical Guideline of Stroke for the United Kingdom (UK) and Ireland, and the 2023 World Stroke Organisation’s synthesis of global stroke guidelines do not include PCOS as a vascular risk-enhancer [13,14]. The 2025 scientific statement from World Stroke Organisation recognises that gender differences shape stroke risk, treatment, care and research. However, it focuses on post-partum and menopause as risk states and does not include PCOS as a risk-enhancing factor [15]. The Sentinel Stroke National Audit Programme (SSNAP) does not include PCOS as a risk-enhancing factor and reveals that we lack nationwide audit metric insights into outcomes or care experiences for women with PCOS who experience stroke [16]. The omission of PCOS s in major cardiovascular and stroke risk prediction tools, including the QRISK3,Essen Stroke Risk Score (ESRS) and the recently developed PREVENT (Predicting Risk of cardiovascular disease EVENTs) model, further highlights this practice gap [17,18,19,20].

Importantly, it remains uncertain whether PCOS confers an independent vascular risk or primarily reflects an aggregation of established cardiometabolic risk factors, including obesity, insulin resistance, dyslipidaemia, and type 2 diabetes. Some epidemiological evidence demonstrates attenuation of vascular risk estimates after adjustment for BMI and metabolic variables, suggesting that the observed association may be partially mediated through these pathways rather than PCOS per se [21,22]. Accordingly, PCOS may be conceptualised both as a potential independent risk enhancer and as a clinical marker identifying women with an adverse cardiometabolic profile. This distinction is critical when interpreting epidemiological data and has possible implications for risk stratification and preventive strategies.

Most stroke units, vascular clinics, and cardiology services do not routinely record reproductive history or recognise PCOS as a formal risk-enhancing factor. This limits opportunities for early identification of high-risk women and the timely initiation of preventive measures during the long preclinical phase of vascular disease. This gap can also be understood through the lens of sex-specific medicine, which emphasises that reproductive, hormonal, and pregnancy-related factors may modify vascular risk in ways not captured by traditional models. Bridging the PCOS evidence-to-practice gap requires longitudinal evidence, validation within risk prediction models, and multidisciplinary collaboration to support clinical implementation.

We present a narrative review that aims to: (1) synthesise current epidemiological, pathophysiological and risk stratification evidence linking PCOS to ischaemic stroke and associated CVD, (2) critically appraise international and national guideline recommendations and major risk prediction tools on ischaemic stroke and cardiovascular risk screening and management in PCOS, and (3) identify practical strategies to bridge the PCOS evidence–practice divide in stroke and cardiovascular medicine.

## 2. Methods (Search Strategy, Inclusion/Exclusion Criteria, Data Collection and Quality Assessment)

We conducted a comprehensive literature search across the databasesMedline, Embase, PubMed, and Google Scholar covering publications from January 2000 to December 2025 using pre-defined keywords including “polycystic ovary syndrome” “PCOS” “ischaemic stroke” “cerebrovascular disease” “stroke” “cardiovascular disease” “vascular” “myocardial infarction” “heart attack” “hypertension” “blood pressure” “dyslipidaemia” “lipid” “risk factors” “score” “scoring systems” “guideline” and “framework”. Terms were combined using Boolean operators (‘AND’, ‘OR’) across databases.

### 2.1. Inclusion Criteria

Inclusion criteria were as follows: studies published in the English language between 2000 and 2025, observational studies, cohort studies, clinical trials, pathophysiological studies, systematic reviews, meta-analyses, guideline recommendations, and consensus statements from major societies. 

The primary population focus was adult women from the age of 18 and post-menopausal women who had been diagnosed with PCOS using either of the following criteria: National Institutes of Health criteria (1990), the Rotterdam criteria (2003), and the Androgen Excess Society definition (2006) [5,6,7].

*Relevant studies had the following outcomes of interest:*

1. Incidence and characterisation of ischaemic stroke in patients with PCOS
2. Incidence and characterisation of cardiovascular disease and its associations, including myocardial infarction, hypertension, aortic disease, arrhythmias, heart valve disease, heart failure, peripheral arterial disease, dyslipidaemia and metabolic syndrome in patients with PCOS.

### 2.2. Exclusion Criteria

Studies were excluded if they did not focus on human subjects and those that did not mention ischaemic stroke or any associated CVD. We excluded non-peer-reviewed articles (such as conference abstracts, letters and editorials), case reports and case series. Additional exclusions included studies published before 2000 to focus on more recent data relevant to PCOS. Non-English studies were excluded for feasibility reasons, which may have introduced language bias and limited capture of relevant data from non-English-speaking settings.

To ensure the robustness of our findings, we prioritised higher-quality evidence including cohort studies, systematic reviews and meta-analyses. We also prioritised studies that controlled for key confounding factors such as age, body mass index (BMI) and menopausal status. We placed an emphasis on studies conducted in a variety of geographical locations to minimise geographical bias.

### 2.3. Quality Assurance

To enhance robustness, we prioritised higher-quality evidence, particularly large cohort studies, systematic reviews, and meta-analyses. Where overlapping datasets or multiple publications from the same cohort were identified, the most comprehensive or recent study was included. This structured approach aimed to provide a balanced, methodologically sound synthesis of current evidence on cerebrovascular risk in women with PCOS.

Given the narrative design and the heterogeneity of included study types, a formal quantitative quality assessment was not performed. Instead, emphasis was placed on higher-quality evidence, including large cohort studies, systematic reviews, and meta-analyses, particularly those adjusting for key confounding variables such as BMI, age, and metabolic comorbidities. The strengths and limitations of the evidence base are addressed qualitatively in the discussion.

## 3. Results

### 3.1. Epidemiology of Ischaemic Stroke and Associated CVD in Women with PCOS

The evidence base included in this review is heterogeneous, comprising a total of 117 references, including large population-based cohort studies, systematic reviews and meta-analyses, mechanistic studies, and international guideline documents. The epidemiological evidence is predominantly derived from observational cohort studies and meta-analyses, with sample sizes ranging from small mechanistic studies to large population datasets exceeding 1 million participants [20,21,22,23,24,25,26].

Study populations largely included reproductive-aged women, with some studies extending into post-menopausal populations [20,21,22,23,24,25,26]. Age ranges varied across studies, typically spanning early adulthood to midlife, reflecting the natural history of PCOS and its long-term cardiometabolic implications [20,21,22,23,24,25,26].

Only a subset of included studies specifically examined ischaemic stroke outcomes, while others evaluated composite cardiovascular endpoints or intermediate cardiometabolic risk factors. This heterogeneity in study design, populations, and outcomes should be considered when interpreting the findings [20,21,22,23,24,25,26].

Current evidence indicates an elevated risk of cerebrovascular disease among women with PCOS [20,21,22,23,24,25,26] (Table 1).

A recent meta-analysis in 2025, which pooled data from 11 stroke studies, reported a statistically significant association between PCOS and increased risk of stroke (Odds Risk (OR): 1.89, 95% Confidence Interval (CI): 1.22–2.55, I^2^ = 97.7%) [20]. Although overall risk increased, its magnitude differed based on study design and population characteristics due to significant heterogeneity. However, all-cause mortality, cardiovascular death, myocardial infarction (MI) and ischaemic heart disease (IHD) were not statistically significantly increased in women with PCOS. These findings contrast with a recent meta-analysis in 2023, which included 346, 486 women and showed that menstrual irregularity/oligomenorrhoea was associated with overall CVD (Relative Risk (RR): 1.29; 95% CI: 1.09–1.53) but no statistically significant increased risk of cerebrovascular disease/stroke was found [13]. Even after adjustment for obesity, these results remained broadly consistent [13].

The increased risk of stroke in women with PCOS has been corroborated by a previous meta-analysis in 2017, which involved a total of 237, 647 patients, and found that PCOS was associated with a 36% increase in the odds of stroke (OR: 1.36; 95% CI, 1.09–1.70) [22]. However, after adjusting for BMI, the association between PCOS and stroke remained increased, but the odds ratio did not reach statistical significance (OR: 1.24; 95% CI, 0.98–1.59) [22]. After adjustment for obesity, these results remained broadly consistent.

A 2023 systematic review and meta-analysis of 1.06 million women demonstrated that PCOS was associated with higher risk of composite CVD (OR: r 1.68 [95% CI, 1.26–2.23]; I^2^ = 71.0%), composite ischemic heart disease (OR:1.48 [95% CI, 1.07–2.05]; I^2^ = 81.0%), myocardial infarction (OR: 2.50 [95% CI, 1.43–4.38]; I^2^ = 83.3%), and stroke (OR: 1.71 [95% CI, 1.20–2.44]; I^2^ = 81.4%). The relationship with cardiovascular mortality was less clear (OR, 1.19 [95% CI, 0.53–2.69]; I^2^= 0%) [23].

A recent analysis of all women diagnosed with PCOS on the Danish nationwide registries from 1995 to 2024 demonstrated that, over a 25-year period, women with PCOS were 50% more likely than those without PCOS to develop stroke and acute myocardial infarction [24]. In a similar vein, an older systematic review and meta-analysis by de Groot et al. (2011) showed that the relative risk for coronary heart disease (CHD) or stroke was increased at 2.02 in women with PCOS compared to those without (95% CI: 1.47–2.76) [25].

The most recent systematic review and meta-analysis analysing the effect of PCOS on ischaemic stroke, hypertension and CVD show that women with PCOS have a statistically significantly increased risk of stroke (pooled RR: 1.28; 95% CI: 1.10–1.49, I^2^: 54.09%), hypertension (pooled RR: 1.45; 95% CI: 1.32–1.61, I^2^: 82.20%) and CVD (pooled RR: 1.41; 95% CI: 1.17–1.71, I^2^: 97.05%) [26]. Independent of BMI, PCOS was still associated with higher risks of stroke and CVD [26].

Taken together, the available studies [20,21,22,23,24,25,26] suggest a signal toward increased cerebrovascular risk in women with PCOS, but effect estimates vary considerably across analyses. This variation likely reflects differences in diagnostic criteria, study populations, outcome definitions, duration of follow-up, and adjustment for obesity and metabolic confounders. The substantial heterogeneity reported across meta-analyses likely reflects variation in PCOS definitions, age and ethnic composition, cardiometabolic burden, and whether estimates were adjusted for BMI, diabetes, or dyslipidaemia.

The observation that stroke associations are more consistent than those for myocardial infarction or mortality may reflect differences in event rates, outcome definitions, duration of follow-up, or the possibility that cerebrovascular risk in PCOS is mediated through mechanisms not fully mirrored by other cardiovascular endpoints [23].

### 3.2. Pathophysiology of PCOS

**Figure 1:**
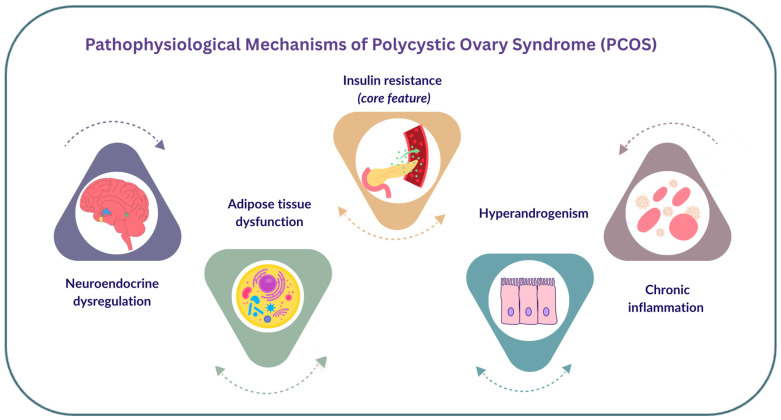
The main pathophysiological mechanisms of PCOS. The depicted pathways are interrelated and overlapping, with arrows indicating their bidirectional and interconnected nature. Insulin resistance is the core feature, interacting with and driving other key mechanisms including neuroendocrine dysregulation, adipose dysfunction, hyperandrogenism, and chronic inflammation. Figures were drawn originally in Canva [27].

The pathophysiological mechanisms linking PCOS to an increased risk of ischaemic stroke and associated CVDs are multi-factorial, driven by a complex interplay between neuroendocrine, epigenetic, genetic and environmental influences that converge to create downstream cerebrovascular and CVD effects, leading to an increased incidence of ischaemic stroke and vascular sequelae as summarised in Figure 1 [27].

The mechanistic evidence presented is derived from a combination of human observational and clinical studies, alongside experimental data from preclinical and in vitro models, with many studies focusing on metabolic and endothelial pathways rather than stroke-specific outcomes (Figure 1).

#### 3.2.1. Neuroendocrine Dysregulation

Women with PCOS exhibit a higher frequency of gonadotropin-releasing hormone pulses (GnRH) pulses [28]. This promotes increased levels of luteinizing hormone (LH) over follicle-stimulating hormone (FSH) [28]. The resultant raised FSH:LH ratios stimulate androgen production from ovarian theca cells, causing anovulation and small antral follicles [28].

Evidence from pre-clinical models suggests that the increasingly small antral follicles secrete anti-Mullerian hormone and further amplify GnRH pulses [29]. High levels of anti-Mullerian hormone in women with PCOS further increase GnRH pulsatility, leading to chronic anovulation and androgen excess [30]. 

Although indirect, these disturbances may contribute to an adverse vascular milieu relevant to stroke risk.

#### 3.2.2. Insulin Resistance

Insulin resistance is the core pathophysiological feature of PCOS [31]. Insulin resistance is present in 94% of obese and 60% of lean women with PCOS [32]. Although the exact mechanisms of insulin resistance remain unclear, Ref. [33] indicates that insulin resistance has been found to impair glucose metabolism in the liver, adipose tissue and skeletal muscles; the ovaries and adrenal glands demonstrate preserved insulin sensitivity [33,34].

Reproductive dysfunction and hyperandrogenism are both driven by compensatory hyperinsulinaemia. This is supported by the observation that disorders of severe insulin resistance such as type A and B insulin resistance syndromes also exhibit PCOS-like phenotypes such as menstrual irregularities and hyperandrogenism [35,36]. Furthermore, in type B insulin resistance, anti-insulin receptor antibodies have been shown to decrease with immunosuppressive therapy, leading to restoration of regular ovulatory cycles and resolution of hyperandrogenism [37]. Complementary evidence from experimental models in mice demonstrates that deletion of the insulin receptor in ovarian theca cells reverses hyperandrogenism and reproductive dysfunction [38]. Hyperandrogenism and infertility are reversed by ovarian-specific deletion of the insulin receptor in theca cells, showing a direct role of insulin in ovarian dysfunction and implying that hyperinsulinaemia may be a trigger for PCOS-like characteristics [38].

By stimulating GNRH1 expression, insulin resistance affects hypothalamic–pituitary signalling. This increases GnRH-driven LH release, which boosts ovarian androgen production and compromises follicular function [33,34].

Furthermore, emerging evidence suggests that abnormalities in glucose regulation, including glycaemic variability and hypoglycaemic episodes, may contribute to broader neurological vulnerability, including neurodegenerative conditions such as Parkinson’s disease, highlighting potential overlap between metabolic and neurological disease pathways [39]. Therefore, it is plausible that women with PCOS are at risk of a range of neurological conditions. Insulin resistance appears to remain a key pathway through which PCOS may increase the risk of cerebrovascular disease later in life.

#### 3.2.3. Adipose Tissue Dysfunction

There is growing evidence that PCOS is characterised by intrinsic adipose tissue malfunction as opposed to abnormal fat distribution. Refs. [40,41,42,43] Compared to their BMI-matched controls, women with PCOS have been found to have larger adipocytes, which correlates with an increased degree of hyperandrogenism [42,43,44]. Experimental studies using in vitro human cells demonstrate that excess androgens impair adipose stem cell differentiation into mature adipocytes, thereby reducing adipogenesis and contributing to adipose dysfunction [45,46].

Furthermore, due to dysregulated lipolysis, there are elevated levels of non-esterified fatty acids which are aberrantly deposited in the pancreas, liver and muscle [41]. This process contributes to chronic inflammation and insulin resistance [47]. These findings have been developed from preclinical models, including prenatally androgen-exposed sheep and non-human primates, suggesting a role for developmental programming in PCOS [47]. A key factor in this process is the increased expression of aldo-ketoreductase type 1C3 (AKR1C3) in the subcutaneous tissue of women with PCOS [48]. The conversion of androstenedione to testosterone is catalysed by AKR1C3. Insulin increases AKR1C3 expression [49,50]. Lastly, adipose tissue dysfunction in PCOS is associated with aberrant adipokine production. A meta-analysis in 2021 revealed that, independent of obesity status, women with PCOS have elevated pro-inflammatory adipokines (such as chemerin, resistin and leptin) alongside significantly reduced adiponectin levels [49,50].

#### 3.2.4. Hyperandrogenism

Up to 60–75% of women with PCOS present with hyperandrogenism [51,52,53]. In 20–30% of cases, the adrenal gland handles androgen excess due to heightened ACTH-mediated steroid response [53]. Insulin resistance and a self-reinforcing cycle of hyperandrogenism are both perpetuated by excess androgen levels, impaired adipogenesis, poorly regulated lipolysis and decreased insulin-sensitising adipokines such as adiponectin [54,55]. Hyperandrogenism may therefore act as a vascular risk amplifier, particularly in metabolically adverse phenotypes.

#### 3.2.5. Chronic Inflammation

Leucocytes, C-reactive protein and pro-inflammatory cytokines are higher in PCOS patients as compared to controls [56]. Even non-obese women with PCOS demonstrate M1 macrophage infiltration in subcutaneous fat tissue. These macrophages release TNF and IL-6, which worsen systemic inflammation [57]. PCOS has been linked to dysregulated complement system pathways, with higher levels of alternative pathway proteins including properdin and C3, particularly in the presence of insulin resistance and obesity [58,59,60]. This reflects a bidirectional relationship in which insulin both results from and drives complement pathway activation [58]. Chronic inflammatory activation provides biological plausibility for later vascular events, including stroke.

#### 3.2.6. The Link to Ischaemic Stroke and Associated Cardiovascular Sequelae

The pathophysiological mechanisms collectively contribute to endothelial dysfunction, accelerated atherosclerosis, and increased thrombotic tendency, which are pivotal mechanisms in both ischaemic stroke and associated cardiovascular pathologies [61]. Specifically, insulin resistance, which is a hallmark feature of PCOS, plays a vital role by promoting systemic inflammation, dyslipidemia, and hypertension, thereby contributing to the development of atherosclerotic plaques and increasing the risk of ischaemic stroke [62]. A 2025 case–control design study revealed that, driven by disrupted levels of nitric oxide, patients with PCOS have a dysregulated balance between vasodilation and vasoconstriction, which contributes to endothelial dysfunction and heightened vascular risk [63]. In the same study, a statistically significant difference was found between women with PCOS and controls in the levels of FSH, LH, testosterone, SHBG and HDL (*p* < 0.05), which may imply a possible correlation between higher levels of sex hormones and the predisposition to endothelial dysfunction in women with PCOS [63].

The heightened oxidative stress and chronic low-grade inflammation prevalent in PCOS patients, shown by elevated levels of inflammatory cytokines, may trigger endothelial cell dysfunction and thrombosis, which are critical precursors to atherogenesis and thrombotic events such as ischaemic stroke [64].

The cumulative effect of insulin resistance, hyperinsulinaemia and dyslipidaemia leads to dysfunction in circulating endothelial progenitor cells, leading to the enhanced susceptibility to vascular events seen in women with PCOS [65]. The interaction between insulin resistance and hyperandrogenism exacerbates dyslipidemia, characterised by elevated triglycerides and low high-density lipoprotein cholesterol, and promotes the development of small, dense low-density lipoprotein particles, which are highly atherogenic [66]. This metabolic milieu, frequently compounded by obesity, predisposes women with PCOS to accelerated plaque formation and vascular remodelling, further elevating their risk of both ischaemic stroke and cardiovascular disease [67].

While these mechanisms provide strong biological plausibility linking PCOS to vascular dysfunction, it is important to note that most evidence derives from metabolic and endothelial studies rather than stroke-specific outcomes. Therefore, these pathways should be interpreted as indirect contributors to vascular risk rather than as evidence of a direct causal relationship between PCOS and ischaemic stroke. Where available, vascular biomarkers and surrogate markers of endothelial dysfunction or subclinical atherosclerosis may provide additional support for biological plausibility, although these remain indirect rather than stroke-specific measures. Further prospective studies with cerebrovascular endpoints are required to establish causal links.

### 3.3. Risk Stratification in PCOS: The Role of Phenotype, Obesity, Ethnicity, Age and Reproductive Factors

The clinical presentation of PCOS is heterogeneous, reflecting different combinations of hyperandrogenism, ovulatory dysfunction, and polycystic ovarian morphology as defined by the Rotterdam criteria. These diagnostic criteria give rise to four distinct phenotypes: A (hyperandrogenism + ovulatory dysfunction + polycystic ovaries), B (hyperandrogenism + ovulatory dysfunction), C (hyperandrogenism + polycystic ovaries), and D (ovulatory dysfunction + polycystic ovaries) [68]. Evidence suggests that women with hyperandrogenic phenotypes (A, B and C) carry the highest cardiometabolic burden and therefore face a greater risk of cardiovascular disease, including potential downstream cerebrovascular risk [69].

Obesity is a major risk amplifier within PCOS, as 38–88% of women with PCOS are either overweight or obese [70] (Table 1). Weight gain worsens insulin resistance, features of metabolic syndrome and hyperandrogenism, each of which independently increases the risk of adverse vascular outcomes, including established risk factors for ischaemic stroke [70]. Importantly, obesity interacts with PCOS phenotype and is a well-documented risk factor for type II diabetes and poor lipid profile [71].

Ethnicity further modifies the cardiometabolic profile of women with PCOS (Table 1). Primarily, the main presentations of racial and ethnic differences in PCOS are hirsutism and metabolic features. South Asian, Hispanic, Middle Eastern and Mediterranean women are more hirsute than white and East Asian women [72,73]. In comparison, East Asian women are less hirsute than white women [72,73]. In terms of cardiometabolic features, black women present with higher BMI, insulin resistance, and blood pressure but lower levels of triglycerides as compared to white women [72,74]. Similarly, Hispanic women present with higher BMI and insulin resistance than white women [72,74]. Although South Asian and East Asian women present with lower BMI, they exhibit higher central adiposity and risk of type II diabetes mellitus compared to white women [72,75]. These differences in cardiometabolic profile may have implications for cerebrovascular risk, although stroke-specific data across ethnic groups remain limited.

A national Danish registry cohort study found that, compared to age-matched controls, women with PCOS have higher CVD risk, particularly prominent among those in their 30–40s [76]. The elevated CVD risk among younger women suggests that PCOS-related CVD risk is more significant in earlier life, which highlights the need for early preventive interventions. Evidence indicates that post-menopausal women with PCOS continue to be at higher risk of coronary artery disease and cerebrovascular disease, including ischaemic stroke, compared to post-menopausal women without PCOS [77]. The main mechanisms that serve to increase the risk of cerebrovascular and cardiovascular disease are cited to be persisting high levels of androgens throughout menopause, maturity-onset diabetes mellitus and obesity [77].

Furthermore, reproductive factors can also act as long-term risk modifiers (Table 1). A retrospective cohort study comprising 1023 women with PCOS revealed that women with PCOS had a higher incidence of pregnancy-induced hypertension (PIH) (10.8% versus 6.6%), including gestational hypertension (5.8% versus 3.6%) and pre-eclampsia or HELLP (haemolysis, elevated liver enzymes, low platelet count) (4.9% versus 3.0%, all *p* < 0.05) [78]. PCOS was associated with higher odds of PIH (OR 1.7, 95% CI 1.2–2.4), which remained statistically significant after adjusting for infertility, nulliparity, age, race and ethnicity [78]. During the peri-partum period, the odds of stroke, MI, spontaneous coronary artery dissection (SCAD) and cardiomyopathy are significantly increased for women with hypertensive disorders of pregnancy versus women without [79,80]. Meta-analyses and large prospective cohort studies have demonstrated a two-fold increase in CVD in later life for women with gestational hypertension or pre-eclampsia [81,82]. Hypertension, hyperlipidaemia and diabetes mellitus are diagnosed up to 10 years earlier in women with a history of hypertensive disorders of pregnancy compared to women without [83,84]. This suggests that women with PCOS are more likely to experience hypertensive disorders in pregnancy, which may contribute to increased long-term risk of ischaemic stroke and broader CVD.

Cardiovascular risk appears highest among hyperandrogenic phenotypes; however, stroke-specific data by phenotype remain limited, and current evidence mainly supports differential cardiometabolic rather than directly established cerebrovascular risk. Ethnic differences should be interpreted cautiously, as ethnicity may interact with socioeconomic conditions, healthcare access, and environmental exposures, and broad ethnic categories risk obscuring important within-group heterogeneity. Taken together, the multi-factorial presentation of PCOS supports a multidimensional risk model in which vascular risk is determined by the interaction among intrinsic features (phenotype, hyperandrogenism), acquired factors (obesity, insulin resistance), demographic modifiers (ethnicity, age), and reproductive history. Rather than acting independently, these variables cluster and interact, producing a spectrum of risk profiles ranging from relatively low-risk lean phenotypes to high-risk individuals with metabolic syndrome and adverse reproductive histories. An integrated framework that includes these features and factors may provide a more clinically useful approach to risk stratification than considering individual factors in isolation.

In summary, the risk of ischaemic stroke and associated cardiovascular disease in PCOS varies across phenotype, BMI, ethnicity, age, and reproductive history (Table 1), although the extent to which these factors independently influence stroke risk remains uncertain. Hyperandrogenic phenotypes, obesity, high-risk ethnic backgrounds, and adverse reproductive events converge to create the most vulnerable subgroups; recognition of this heterogeneity is crucial for targeted prevention and individualised management strategies [85,86,87,88,89].

**Table 1:** Summary of the principal epidemiological and risk-stratification findings which highlights the variation in effect estimates, outcome types, and modifiers of risk across studies.

Domain	Study Type/Population	Key Findings	Effect Size/Estimates	Reference
Stroke risk in PCOS	Meta-analysis (11 studies, 2025) Meta-analysis (systematic review)	PCOS associated with increased ischaemic stroke risk Increased stroke risk, partially attenuated after BMI adjustment	OR ^1^ 1.89 (95% CI 1.22–2.55) OR ^1^ range 1.28–1.36	[13,20,21,22,23,24]
Cardiovascular events	Nationwide registry cohort (Denmark, 25-year follow-up)	Increased incidence of ischaemic stroke and myocardial infarction in PCOS	Stroke HR ^2^ ~1.3; MI HR ^2^ ~1.2	[25]
Cardiometabolic risk	Observational cohorts	Earlier onset of hypertension, dyslipidaemia, and diabetes in PCOS	Up to 10 years earlier than controls	[26,27,28]
Pregnancy-related stroke risk	Retrospective cohort (n = 1023 PCOS pregnancies)	Higher incidence of pregnancy-induced hypertension and pre-eclampsia	PIH ^3^ 10.8% vs. 6.6%; OR ^1^ 1.7 (95% CI ^4^ 1.2–2.4)	[78,79,80,81]
	Meta-analyses and large cohorts	Hypertensive disorders of pregnancy increase later stroke and CVD risk	~2-fold increase in later-life CVD	
Phenotype-specific risk	Observational studies	Hyperandrogenic PCOS phenotypes associated with higher metabolic risk	Greater insulin resistance, dyslipidaemia	[32,33,34,35]
Obesity-independent risk	Adjusted cohort analyses	Stroke risk persists after BMI adjustment	Significant residual risk	[22,24]
Sex- and gender-specific stroke policy	World Stroke Organization Scientific Statement (2025)	Recognition of pregnancy, postpartum, and menopause as stroke risk modifiers; PCOS not listed	Qualitative	[16]

^1^ OR: Odds ratio, ^2^ HR: hazard risk, ^3^ PIH: pregnancy-induced hypertension, ^4^ CI: confidence interval.

## 4. Discussion

This review examines the relationship between PCOS, ischaemic stroke risk and associated CVD, integrating epidemiological evidence with pathophysiological insights. Our results suggest a modest-to-moderate association of PCOS with ischaemic stroke, but interpretation is limited by heterogeneity and residual confounding [20,21,22,23,24].

Although the available epidemiological literature suggests an association between PCOS and cerebrovascular and cardiovascular disease, the current evidence base does not support a direct causal relationship. Much of the existing evidence is derived from observational studies, which are inherently susceptible to residual confounding, particularly by adiposity, metabolic comorbidities, and variations in PCOS phenotyping. Association alone does not establish causality, and causal inference requires stronger evidence, ideally supported by consistent findings across study designs and frameworks such as the Bradford Hill criteria. Although much of the aforementioned evidence relates to overall cardiovascular risk, these factors are also relevant to stroke prevention because they cluster around established cerebrovascular risk pathways, particularly hypertension, diabetes, and obesity [20,21,22,23,24,25,26].

Some meta-analytic data reveal an up to 89% increased risk of stroke (OR 1.89 (95% CI 1.22–2.55) and up to 68% increased risk of composite CVD, although heterogeneity was high (I^2^ = 97.7%), indicating important variation in study design, populations, and confounder adjustment [23]. Women with hyperandrogenic phenotypes (A, B and C) carry the highest cardiometabolic burden [69]. Obesity interacts with PCOS and confers increased risk of developing type II diabetes and poor lipid profile [71]. Ethnicity further modifies the cardiometabolic profile of women with PCOS, with black women and Hispanic women presenting with higher BMI and insulin resistance compared to white women [72,74].

The cumulative effect of insulin resistance, hyperinsulinaemia and dyslipidaemia leads to dysfunction in circulating endothelial progenitor cells, leading to the enhanced susceptibility to vascular events seen in women with PCOS [65]. Driven by disrupted levels of nitric oxide, patients with PCOS have a dysregulated balance between vasodilation and vasoconstriction, which contributes to endothelial dysfunction and heightened cardiovascular risk [63]. A statistically significant difference was found between women with PCOS and controls in the levels of FSH, LH, testosterone, SHBG and HDL (*p* < 0.05), which may imply a possible correlation between higher levels of sex hormones and the predisposition to endothelial dysfunction in women with PCOS [63].

The interpretation of pooled estimates included in this review must be approached with caution, given the substantial heterogeneity observed across studies, with some I^2^ values exceeding 90%, reflecting differences in diagnostic criteria, study populations, follow-up duration, and adjustment for confounders [23]. Importantly, some analyses demonstrate attenuation or loss of statistical significance after adjustment for BMI and metabolic variables, indicating that cardiometabolic factors may substantially mediate the observed association [22]. These findings raise the possibility that PCOS-related vascular risk may be largely indirect, operating through established pathways such as insulin resistance, obesity, and dyslipidaemia, rather than representing a consistently independent cerebrovascular risk factor. Accordingly, the magnitude and independence of stroke risk attributable to PCOS remain uncertain. Clinically, this heterogeneity supports a more individualised approach to vascular risk assessment in PCOS, with greater attention to phenotype, obesity, metabolic profile, reproductive history, and social context rather than assuming uniform risk across all women with PCOS.

The recognition of PCOS as a vascular risk-enhancing factor has implications in the development of preventative measures against ischaemic stroke and CVD in women. Specialty-specific guidelines in endocrinology, cardiology, obstetrics and gynaecology increasingly acknowledge the heightened vascular burden associated with PCOS (Table 2). Notably, the 2023 International Evidence-based Guideline for the Assessment and Management of PCOS, along with its American and Nordic adaptations, recognises that women with PCOS have an increased burden of cardiovascular risk factors and may be at higher risk of cardiovascular disease (Table 2). Meta-analytical evidence cited within the guideline includes increased odds of outcomes such as ischaemic stroke, myocardial infarction, and composite cardiovascular disease; however, the strength of the evidence is rated as low to very low, as most of the data is derived from observational studies [90,91,92,93,94,95,96,97]. Currently, the Royal College of Physicians’ National Clinical Guidelines for Stroke (UK and Ireland, 2023) and the World Health Organisation’s 2023 synthesis of global stroke guidelines do not include PCOS within recognised vascular risk factors [14,15,98,99] (Table 2).

Widely used vascular risk prediction and stratification tools implemented in routine clinical practice such as the ABCD^2^ score for transient ischaemic attack (TIA) risk, the QRISK3 score, the Essen Stroke Risk Score (ESRS), the Framingham risk score for hard coronary heart disease, the PREVENT (Predicting Risk of Cardiovascular Disease Events) model and the Assessing cardiovascular risk using Scottish Intercollegiate Guidelines Network to assign preventive treatment (ASSIGN v 2.0) do not include PCOS as a variable [19,100,101,102,103,104,105] (Table 3).

The Reynolds Risk Score represents a seminal example of a female-specific risk screening tool [106] (Table 3). Derived from a prospective cohort of over 24,000 healthy women aged ≥ 45 years, who were followed for 10 years for incident cardiovascular events, including MI and ischemic stroke [106], the model utilised Cox proportional hazards modelling and Bayesian Information Criterion to identify both traditional predictors (age, blood pressure, smoking, lipids) and novel factors such as high-sensitivity C-reactive protein and parental history of premature MI [106]. Subsequent validation analyses demonstrated improved discrimination, calibration and reclassification of women into more accurate risk strategies compared with traditional models [107]. These findings underscore the value of incorporating sex-specific predictors into vascular risk algorithms and support the rationale for further refinement of female-specific models [107,108].

The updated QRISK4 model incorporates female-specific risk factors such as pre-eclampsia and post-natal depression which represents progress in addressing sex-specific vascular risk [109] (Table 3). Similarly, emerging approaches such as the Canadian Heart & Stroke Risk screen tool account for reproductive life events, including pregnancy-related complications and menopause, reflecting progress toward more inclusive and biologically informed risk assessment frameworks [110].

A growing body of evidence indicates that female-specific risk factors such as PCOS, menopausal and perimenopausal status, early menopause, years since menopause, hypertensive disorders of pregnancy, or use of any oestrogen-containing contraceptives are each associated with increased risk of adverse cerebrovascular outcomes [83,85,86]. Updating existing scoring systems or developing novel risk prediction models that incorporate these female-specific variables may enhance the precision and clinical utility of vascular risk assessment of women within these groups [87]. This knowledge has not yet been translated into routine clinical practice in stroke units, cardiology clinics, or vascular services. For stroke, cardiovascular and primary care physicians, addressing this practice gap is crucial for timely risk assessment, targeted preventive strategies, and improved possible outcomes for women with PCOS who may otherwise be overlooked in traditional vascular care pathways.

Although the absence of PCOS from major risk prediction tools highlights a practice gap, there is currently no evidence demonstrating that inclusion of PCOS improves model discrimination, calibration, or clinical utility. At present, the incorporation of PCOS into risk algorithms remains hypothetical and requires validation through prospective modelling studies. Future research should evaluate whether PCOS provides incremental predictive value beyond established cardiometabolic variables.

PCOS disproportionately affects younger women, those from high-risk ethnic backgrounds (as they are at increased risk of higher lipid concentrations and free androgen index compared to white women), and those facing structural barriers to accessing regular healthcare [88]. These populations are frequently underrepresented in epidemiological cohorts, leading to systematic underestimation of risk and poorly captured outcomes [89]. The omission of PCOS therefore compounds pre-existing disparities in women’s healthcare.

The emerging evidence suggests that future updates of stroke and cardiovascular prevention guidelines should consider whether PCOS should be more explicitly recognised as an adverse vascular risk factor, while acknowledging that the current certainty of evidence for clinical endpoints remains limited. However, before PCOS can be formally incorporated into cardiovascular, stroke, and endocrine guidelines as a vascular risk factor, several evidence gaps must be addressed. Current data are predominantly observational and heterogeneous, and it remains unclear whether PCOS confers risk independently or primarily reflects underlying cardiometabolic dysfunction.

**Figure 2:**
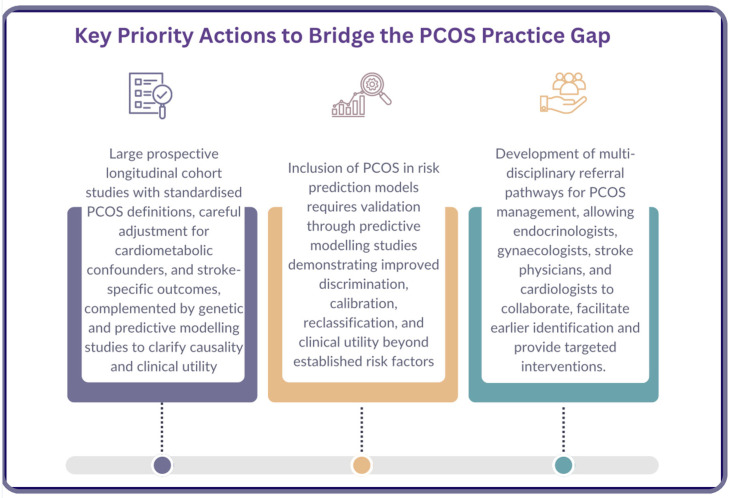
Key priority future actions to bridge the polycystic ovary syndrome (PCOS) practice gap. Figure was drawn originally in Canva [27].

Bridging the PCOS-related practice gap requires a structured and staged approach, progressing from evidence generation to clinical implementation as demonstrated in Figure 2 [27].

Stage 1: Establishing causal and independent risk. Robust prospective research is needed to determine whether PCOS represents an independent vascular risk factor. This includes large, well-designed longitudinal studies with standardised diagnostic criteria, long-term follow-up for stroke-specific outcomes, and rigorous adjustment for cardiometabolic confounders. Complementary approaches, including individual participant data meta-analyses, Mendelian randomisation studies, and predictive modelling analyses, will be essential to clarify causality and clinical relevance. While randomised controlled trials may provide supportive evidence regarding risk modification, they cannot establish independence of risk.

Stage 2: Evaluating clinical utility within risk prediction models. Before PCOS can be incorporated into widely used risk prediction tools, dedicated modelling studies are required to assess its incremental value beyond established risk factors. This includes comparison of model performance with and without PCOS using measures of discrimination, calibration, and reclassification, alongside external validation across diverse populations. Evaluation of clinical impact is also necessary to determine whether inclusion meaningfully alters risk stratification or management decisions. Together, these steps determine whether PCOS provides sufficient predictive value to justify integration into clinical tools.

Stage 3: Translation into clinical practice and implementation in real-world care: Any future clinical framework should account for structural disparities in healthcare access, delayed diagnosis, and uneven cardiometabolic screening, which may disproportionately affect socioeconomically disadvantaged and minority populations. Evidence is also required to demonstrate that recognising PCOS as a vascular risk modifier leads to meaningful improvements in clinical management and stroke-related outcomes. Until such data are available, integration into guidelines and risk prediction tools should be approached with caution.

The principal strength of this review lies in its identification of a critical practice gap linking PCOS to ischaemic stroke, which is infrequently addressed in the existing literature compared to broader CVD outcomes. This review synthesises and critically contrasts major British and international guidelines on spanning ischaemic stroke, cardiovascular disease, gynaecology and endocrinology. Several limitations warrant consideration. As a narrative review, study selection may be subject to selection bias with omission of relevant studies. As complete search strings were not retained, reproducibility of the search strategy may be limited. Although the association between PCOS and composite cardiovascular disease outcomes is well established, relatively few studies report stroke-specific, disaggregated outcomes. This paucity of ischaemic stroke-focused data limits the ability to comprehensively characterise the distinct pathophysiological mechanisms and clinical manifestations linking PCOS to ischaemic stroke. Additional limitations include restrictions to English-language publications which may introduce language bias, and exclusion of grey literature which may have limited inclusion of emerging or unpublished data. Variability in PCOS diagnostic criteria and cardiovascular outcome definitions across studies may have contributed to heterogeneity in reported findings. The current evidence base is largely derived from observational studies and meta-analyses, which are inherently limited by residual confounding and cannot establish causality. Considerable heterogeneity exists in diagnostic criteria for PCOS, contributing to variability in study populations and risk estimates. Stroke incidence in reproductive-aged women remains relatively low, limiting statistical power in many studies and increasing uncertainty in effect estimates. Many studies report composite cardiovascular outcomes rather than stroke-specific endpoints, complicating the interpretation of cerebrovascular risk. Residual confounding remains a major limitation, particularly given incomplete adjustment for obesity, insulin resistance, diabetes, and socioeconomic determinants in some studies. Socioeconomic factors, healthcare access, and ethnicity may further influence observed associations but are inconsistently accounted for across studies. Prospective longitudinal studies with well-characterised PCOS phenotypes and stroke-specific outcomes are needed to clarify the magnitude and independence of risk. These limitations underscore the need for prospective, standardised, and outcome-specific studies before PCOS can be reliably incorporated into guideline frameworks or risk prediction models.

**Table 3:** Major risk scoring systems for stroke and cardiovascular disease.

Author, Year	Scoring System	Geographic Validation	Purpose of Scoring System	Does It Include PCOS or Any Female-Specific Risk Factor?	Risk Factors Included in the Scoring System
Johnston, 2007 [100]	ABCD^2^	United States of America (USA), United Kingdom (UK)	Estimates the risk of stroke after a suspected transient ischaemic attack (TIA)	No	Age ≥ 60 years BP 140/90 Clinical features of TIA (unilateral weakness/speech disturbance without weakness/other symptoms) Duration of symptoms History of diabetes
Weimar, 2008 [19]	Essen Stroke Risk Score (ESRS)	44 countries across six major regions: Latin America, North America, Europe, Asia, the Middle East and Australia	Predicts the 1-year risk of recurrent (fatal and non-fatal) stroke	No	Age Hypertension Diabetes mellitus Previous MI Other CVD except MI Peripheral artery disease Smoker Previous TIA/stroke
Wilson, 1998 [101]	Framingham Score	USA	Estimates 10-year risk of heart attack in patients 30–79 years with no history of coronary heart disease or disease	No	Age Gender Smoker Total cholesterol HDL cholesterol Systolic blood pressure (BP) Blood pressure being treated with medicines
Khan, 2024 [102]	Predicting Risk of Cardiovascular Disease EVENTs (PREVENT) score	40 countries across five major regions: South America, North America, Europe, Asia and Australia	Predicts 10-year and 30-year risk of CVD and CVD sub-types in patients aged 30–79 without known CVD	No	Age Gender Total cholesterol HDL cholesterol Systolic blood pressure Diabetes Current smoker eGFR Using anti-hypertensive medication Using statins BMI Urine albumin-creatinine ratio HbA1c Zip code (social deprivation index)
Goff, 2013 [103]	Atherosclerotic Cardiovascular Disease (ASCVD) Risk Algorithm	USA	Determines 10-year risk of hard ASCVD in patients aged 40–79 years: myocardial infarction, stroke or death due to coronary heart disease or stroke	No	Age Diabetes Sex Smoker Total Cholesterol HDL Cholesterol Systolic BP Treatment for hypertension Race (white, African American, Other)
Welsh, 2025 [104]	Assessing cardiovascular risk using SIGN to assign preventive treatment (ASSIGN v2.0)	Scotland	Estimates 10-year risk of developing coronary heart disease, stroke, transient ischaemic attack, in individuals with no current CVD	No	Age Sex Postcode or Scottish Index of Multiple Deprivation Parent/sibling with coronary heart disease/stroke Diabetes Smoker Systolic BP Total Cholesterol HDL Cholesterol
Hippisley-Cox, 2017 [105]	QRISK3 score	England	Calculates a patient’s risk of developing a stroke or heart attack over the next 10 years (assuming they have no history of CVD and are not on any statins).	No	Age Gender Ethnicity UK Postcode Smoking status Diabetes status Angina or heart attack in a relative < 60? Chronic kidney disease Atrial fibrillation On blood pressure treatment History of migraines Rheumatoid arthritis Systemic lupus erythematosus (SLE) Severe mental illness On atypical antipsychotic medication Regular steroid tablets Erectile dysfunction Cholesterol: HDL ratio value Systolic blood pressure Standard deviation of at least two most recent systolic blood pressure readings BMI
Ridker, 2007 [106]	Reynolds Risk Score for Cardiovascular risk in women	USA	Estimates 10-year cardiovascular risk in women over the age of 45, including myocardial infarction, ischaemic stroke, coronary revascularisation and CVD death.	No	Age Systolic BP Diabetes Current smoker HDL Cholesterol Total Cholesterol High-sensitivity C-reactive protein Parent with MI before the age of 60
Hageman, 2021 [108]	Systematic Coronary Risk Evaluation 2 (SCORE 2)	Europe	Predicts 10-year CVD risk in patients without prior CVD or diabetes- only to be used in European patients aged 40–69 years	No	Sex Age Smoking (Other, Current) SBP Total Cholesterol HDL Cholesterol Risk region (map provided underneath score with developed countries generally scoring low risk-moderate risk and developing countries generally scoring high risk—very high risk)
Hageman, 2021 [108]	Systematic Coronary Risk Evaluation—Older Persons (SCORE2-OP)	Europe	Predicts 10-year CVD risk in patients aged 70 or older without known CVD		As SCORE2 factors Diabetes
Hippisley-Cox (2024) [109]	QR4	UK	Predicts 10-year risk of CVD and stroke	Yes	As QRISK3 factors Learning disability Down Syndrome Chronic Obstructive Pulmonary Disease (COPD) Lung cancer Oral cancer Blood cancer Brain cancer Pre-eclampsia Post-natal depression
Heart & Stroke Canada (2025) [110]	Heart and Stroke Risk Screen Tool	Canada	Personalised risk profile and action plan	Yes	Sex Age Past medical history Lifestyle behaviours Pregnancy Menopause

### Management

Given the limited stroke-specific evidence base, management recommendations in women with PCOS should currently be framed as extrapolated risk-reduction principles rather than PCOS-specific stroke prevention strategies. Reducing the risk of ischaemic stroke and associated CVD in women with PCOS requires a multidisciplinary structured and individualised approach that targets modifiable vascular risk factors. A thorough gynaecological history should be taken and recorded clearly in the patient’s notes. In clinical practice, it is important that physicians enquire about gender identity, as transgender men may warrant specific screening due to evidence suggesting an increased risk of PCOS as compared to the general population [111]. Transgender men receiving long-term androgen therapy should be assessed for features of PCOS and insulin resistance, since both PCOS and exogenous androgen exposure have been reported as independent risk factors for the development of diabetes [112].

Patients with PCOS should be informed early (ideally at the time of diagnosis) about their increased long-term risk of ischaemic stroke, hypertension, type 2 diabetes mellitus (T2DM), and other forms of CVD. Early counselling provides an opportunity to initiate preventive strategies, optimise adherence to follow-up, and reinforce the importance of regular monitoring and treatment. This role is essential in primary care, where most women with PCOS are diagnosed and monitored for weight, blood pressure, glycaemia, and lipid profiles.

Lifestyle modification forms the cornerstone of prevention. All women with PCOS should be encouraged to adopt a healthy diet and engage in regular physical activity [113]. Weight should be monitored every 6–12 months, with more frequent assessment where appropriate, following shared decision-making with the patient [98]. In women who are overweight or obese, weight reduction should be prioritised, as it has been shown to improve hyperandrogenism, insulin resistance, menstrual irregularities, and to reduce the risk of T2DM and vascular disease [114].

Blood pressure should be measured at least annually, with hypertension managed in accordance with established national guidelines such as those from NICE [98]. A fasting lipid profile, including total cholesterol, LDL cholesterol, HDL cholesterol, and triglycerides, should be obtained at the time of diagnosis, irrespective of BMI or age and repeated as clinically indicated [98]. This is particularly important as up to 70% of women with PCOS have dyslipidaemia. Women identified with dyslipidaemia should be referred for specialist management, and statin therapy initiated when appropriate [98]. Statin therapy may offer therapeutic benefits to women with PCOS, not only by reducing CVD risk and improving insulin resistance, but also through potential effects on steroidogenesis [115]. By limiting the availability of cholesterol, which is a key substrate in steroidogenesis, statins may help modulate enzymatic activity involved in androgen production [115]. Furthermore, statins contribute to anti-inflammatory effects, which may further contribute to improved endocrine and metabolic outcomes in this population [115].

All women with PCOS should undergo baseline glycaemic status testing, with repeat assessment every 1–3 years depending on the presence of risk factors such as elevated BMI [98]. Recommended tests include a 2 h 75 g oral glucose tolerance test (OGTT), fasting plasma glucose, and HbA1c [98]. Women with impaired fasting glucose (6.1–6.9 mmol/L) or impaired glucose tolerance (plasma glucose ≥7.8 mmol/L but <11.1 mmol/L following OGTT) should be offered annual OGTT [98]. Metformin may be considered in women with PCOS to improve insulin sensitivity, mitigate glycaemic progression, achieve menstrual regularity and manage androgen levels, particularly in those who are insulin resistant and/or obese [116]. Evidence for medical interventions in PCOS largely relates to metabolic and cardiovascular risk factor modification rather than direct stroke prevention, and their role should therefore be interpreted cautiously [116]. Smoking status should be reviewed routinely, with structured cessation support offered where appropriate [98].

A combined oral contraceptive (COC) pill is frequently offered to women with PCOS to manage clinical symptoms such as acne and prolonged amenorrhoea. Given the elevated vascular risk in women with PCOS, particularly in women with concurrent metabolic syndrome, COC pill counselling must carefully balance symptom control with safety. Patients should be counselled about the increased vascular risk associated with COC pills, as COC users have been found to have a 1.6-fold increased risk of ischaemic stroke and myocardial infarction [85]. In particular, patients who may suffer from migraine with aura should not be prescribed combined hormonal contraceptives (oestrogen-containing preparations such as the combined oral pill, transdermal patch, or vaginal ring), as these agents confer a clinically significant increased risk of ischaemic stroke [117]. For clarity, contraceptive considerations should be separated into baseline vascular risk assessment and subsequent prescribing implications.

## 5. Conclusions

Polycystic ovary syndrome (PCOS) is a prevalent endocrine disorder with significant vascular consequences. Epidemiological and meta-analytic evidence links PCOS to increased risk of vascular disease, including ischaemic stroke and associated CVD, likely driven by insulin resistance, dyslipidaemia, hypertension, hyperandrogenism, and chronic inflammation. The increased vascular risk is not uniform, being greatest among hyperandrogenic phenotypes, women with obesity, adverse reproductive histories, and high-risk ethnic groups.

PCOS currently remains absent from some stroke and cardiovascular guidelines and is not included in widely used risk prediction models. This creates an evidence-to-practice gap whereby women with PCOS, though at elevated vascular risk, may be classified as low risk in routine care. This may lead to possible clinical consequences, such as women with PCOS presenting with preventable stroke or CVD. There remains a clear need for adequately powered randomised controlled trials to determine whether targeted management of PCOS-related metabolic and hormonal abnormalities results in a measurable reduction in cerebrovascular and cardiovascular events.

The most immediate priorities are prospective studies clarifying the independence of vascular risk in PCOS, predictive modelling studies evaluating whether PCOS improves risk stratification, and development of multidisciplinary care pathways for women with clustered metabolic and reproductive risk factors. This is also a health equity issue, as failure to recognise sex-specific and socially patterned risk modifiers may contribute to delayed identification and preventive care in already underserved groups. Bridging the PCOS evidence-to-practice gap is a clinical imperative, ensuring that women with PCOS are included in ischaemic stroke and CVD prevention strategies.

## Figures and Tables

**Table 2:** National and international guidelines on ischaemic stroke, cardiovascular disease and polycystic ovary syndrome (PCOS).

Guideline	Risk Recognition	Quantified Risks	Screening & Prevention Recommendations	Implementation/Stroke Notes
International Evidence-based PCOS Guideline (2023; developed by (1) The Centre for Research Excellence in Women’s Health in Reproductive Life, part of Monash University, (2) the European Society of Human Reproduction and Embryology, (3) the Endocrine Society and (4) American Society for Reproductive Medicine, endorsed by Royal College of Obstetrics and Gynaecology [90]	PCOS recognised as independent CVD risk factor, including ischaemic stroke	Composite CVD: OR 1.68 (95% CI 1.26–2.23); IHD: OR 1.48 (95% CI 1.07–2.05); MI: OR 2.50 (95% CI 1.43–4.38); Stroke (mainly ischaemic): OR 1.71 (95% CI 1.20–2.44); CVD mortality: OR 1.19 (95% CI 0.53–2.69)	Lipid profile at diagnosis; repeat periodically. Annual BP checks. Oral glucose tolerance test (OGTT) at baseline and with risk change. Consider ethnicity and age.	Explicit call to integrate PCOS into CVD/stroke prevention; practice gap persists.
Acta Obstetricia et Gynecologica Scandinavica-2024 [91]	Endorses international guideline; PCOS as independent CVD risk factor	Same pooled estimates as above	Lipid profile at diagnosis, annual BP, OGTT baseline and periodic, age/ethnicity-adjusted.	System-wide integration urged into Nordic prevention pathways.
US Guidance (American College of Obstetrics and Gynaecologists (ACOG), Practice Bulletins 2018, the Endocrine Society Clinical Practice Guidelines, the Androgen-Excess PCOS Consensus statement [92,93,94]	PCOS carries “substantial metabolic sequelae”; treated as ASCVD “risk-enhancing factor”	Stroke: OR ~1.7× CVD: OR 1.68; IHD: OR 1.48; MI: OR 2.50	Lipid profile (ACOG), BP monitoring (ACOG/Endocrine Society), glucose/OGTT (Endocrine Society); use atherosclerotic cardiovascular disease risk calculators.	Emphasises aggressive risk-factor modification; PCOS not always listed but recognised as female-specific risk enhancer.
2024 Guideline for the primary prevention of stroke: American Heart Association/American Stroke Association [95]	PCOS is a female-specific risk-enhancing factor for diabetes/pre-diabetes	N/A	Screen for diabetes and pre-diabetes.	Quotes American Diabetes Association recommendation to screen women with PCOS for diabetes/pre-diabetes.
European Society of Cardiology; Management of Dyslipidaemias 2019 [96]	PCOS is female-specific risk modifier; risk underestimated in young women	Draws on pooled international data (stroke OR 1.71, MI OR 2.50)	Screen BP, lipids, glucose/HbA1c, BMI; routine follow-up; treat risk per global CVD risk.	Standard stroke prevention; no PCOS-only interventions.
European Society of Hypertension, 2023 [97]	PCOS linked with higher long-term risk of CVD and HTN prevalence	Not quantified separately	Routine BP screening, consider ABPM/HBPM; treat as per standard.	Reports that many women are not aware of increased risk of CVD/HTN due to insufficient screening in younger women, low socio-economic groups and ethnic minorities.
Royal College of Obstetrics and Gynaecologists (UK, Green-top statements): Long-term consequences of Polycystic Ovary Syndrome [87]	Screens for HTN, dyslipidaemia, impaired glucose tolerance in PCOS; no specific link to stroke risk	Reports lifetime risk of CVD is higher in women with PCOS, does not list specific figures	Assess individual CVD risk factors: obesity, exercise, smoking, family history of type II diabetes, hypertension, dyslipidaemia, impaired glucose tolerance, type II diabetes.	No stroke-specific measures. Lipid-lowering treatment is not recommended for hyperandrogenamia and should only be prescribed by a specialist.
National Clinical Guideline for Stroke for the UK and Ireland (2023), endorsed by the Royal College of Physicians [13]	No mention of PCOS or hormonal disorders	N/A	N/A	N/A
National Institute of Clinical Excellence guidelines (NICE, UK) March 2025 [98]	PCOS recognised as risk enhancer for type II diabetes and CVD, advised to use QRISK3 score; stroke not mentioned	Not quantified separately	Measure BMI, BP, lipid levels, OGTT including annual OGTT if applicable. Advise about smoking.	N/A
Scottish Intercollegiate Guidelines Network (SIGN) [13]	PCOS recognised as a cardiometabolic risk factor	As per international data	Routine metabolic + BP monitoring; lifestyle-first.	No PCOS-specific stroke measures.
WHO (Guidelines for Management of Stroke) Updated in 2016 [99]	No mention of PCOS or hormonal disorders	N/A	N/A	N/A
World Stroke Organisation (2023) [14] World Stroke Organisation Scientific Statement (October 2025) [16]	No mention of PCOS or hormonal disorders	N/A	N/A	N/A

## Data Availability

No new data were created or analysed in this study. Data sharing is not applicable to this article.
